# Nanoparticles in Clinical Translation for Cancer Therapy

**DOI:** 10.3390/ijms23031685

**Published:** 2022-02-01

**Authors:** Deepa Mundekkad, William C. Cho

**Affiliations:** 1 Centre for NanoBioTechnology (CNBT), Vellore Institute of Technology, Vellore 632014, Tamil Nadu, India; deepamundekkad@gmail.com; 2 Department of Clinical Oncology, Queen Elizabeth Hospital, Kowloon, Hong Kong, China

**Keywords:** nanoparticles, cancer therapy, nanodrugs, nanochemotherapy, nanomedicine

## Abstract

The advent of cancer therapeutics brought a paradigm shift from conventional therapy to precision medicine. The new therapeutic modalities accomplished through the properties of nanomaterials have extended their scope in cancer therapy beyond conventional drug delivery. Nanoparticles can be channeled in cancer therapy to encapsulate active pharmaceutical ingredients and deliver them to the tumor site in a more efficient manner. This review enumerates various types of nanoparticles that have entered clinical trials for cancer treatment. The obstacles in the journey of nanodrug from clinic to market are reviewed. Furthermore, the latest developments in using nanoparticles in cancer therapy are also highlighted.

## 1. Introduction

Cancer nanomedicine is a fast-advancing field which employed nanoparticles to diagnose and treat cancer [1]. The nanoparticles are capable of delivering the normally insoluble drugs to local and distant tumor sites in a better way, thus reducing the systemic side effects that are generally associated with conventional drug therapies. These nanodrugs are invariably biocompatible, non-immunogenic, non-toxic, and biodegradable, which in turn reduces the risk of unpredicted loss of function or adverse effects encountered in the traditional therapy [2]. The flexibility of nanoparticles in terms of size, shape, selective binding capacity, high permeability and retention effect, surface modification, etc. placed them in a good position in cancer therapy, especially in ovarian, breast, and non-small cell lung cancers [3]. State-of-the-art designs and approaches involving interaction between nanodrugs and the receptors on immune cells (like antigen-presenting cells) are further exploited in cancer therapy for sustained anti-tumor effect [4]. Some pharmaceutical industries underwent a quintessential change with the advent of nanodrugs in cancer therapy.

A more realistic approach to cancer therapy was achieved when the flexibility of nanoparticles in terms of shape, charge, stability and selective binding capacity, inspired the designing of new drugs for therapeutic purposes. Rod-shaped nanoparticles are favored to other shapes for endosomal uptake; positively charged nanoparticles evoke a greater immune response when compared to negatively charged or neutral nanoparticles [5]. Similarly, when the delivery performance of nanocarriers of various shapes and sizes were compared, the short, tubular, non-rigid nanocarrier was found to have better intratumoral invasion capacity [6]. Targeted drug delivery was improved substantially due to the selective binding of nanoligands carrying the drug to the receptors found on the surface of tumor cells. Coupling the nanoparticles with drug-carrying polymers (like poly lactic-co-glycolic acid—PLGA) enhanced the absorption of the drug and induced more toxicity to the tumor cells [7].

Chemoresistance exhibited by cancer cells is one of the reasons why traditional therapy is not effective in combating cancer. Genomic instability within the tumor may contribute to heterogeneity among cancers, which in turn, drives chemoresistance [8]. It is a challenge to deliver the drugs to those sites of temporal or spatial heterogeneity in the wake of chemoresistance. Non-genetic variants, like the mechanisms that the cells adopt to dodge the immune system, are also responsible for resistance to therapy. A combination of different treatment modalities has been successfully employed to overcome therapeutic resistance in cancer cells. Immunotherapy is effective to an extent in combating chemoresistance in cancer as they target the biomarkers for their line of attack. Certain cancer patients are benefited from different modes of cancer immunotherapy [9], cancer vaccines [10], checkpoint inhibitors [11], chimeric antigen receptor (CAR) T-cell therapy [12], cytokines [13], and oncolytic viruses [14]. 

The success of immunotherapy quite depends on how the drug acts on the immune system and promptly activates one or the other component of the immune system to bring about the eradication of cancer cells. Nanoparticles can be fabricated to influence immunomodulation by interacting with the components of immune system [15,16] and thus bring about killing of cancer cells. Nanoparticles have made an amazing impact in nanochemotherapy as is evident from the huge number of nanoparticle-based drugs that have entered clinical trials for cancer therapy. Here, the review centers around the ways that the nanoparticles made a front-line entry into cancer therapeutics over the recent years.

## 2. Nanoparticles in the Treatment of Cancer

There is relentless work going on the world over towards achieving an effective alternative to chemotherapy and a better cure for cancer. Though efforts to treat cancer and improve the efficacy of drugs through nanotechnology are at the research or development stage, nanoparticles have been extensively used in biomedical applications. Due to their nano-size [17] and very special properties (like mass density [18], surface charge [19], etc.) nanoparticles have the advantage of relatively large surface area which facilitates their functionalization with various ligands like DNA [20], RNA [21], peptides [22], aptamers [23], antibodies [24], etc. This will assist in the direct delivery of the modified nanoparticle to the site of action (in vivo). This property greatly influences the development of a wider repertoire of ingredients with active theranostic properties to be incorporated with nanoparticles thus enhancing the pharmacokinetic property (and in turn, the efficacy) of the nanoparticle for cancer treatment [25]. The role of nanoparticles as an immunogenic cargo is also being investigated in traditional radio- and chemo-therapies as well as the advanced adjuvant therapy [26]. The biocompatibility of nanoparticles promoted the development of innovative nanostructures whereby they are now being engaged in totally unconventional roles (as artificial antigen-presenting cells (aAPCs) or as in vivo repository of immunostimulatory molecules) for sustained antitumor activity [27]. aAPCs represent a novel technology in cancer immunotherapy where a nanoparticle-based system mimics the antigen-presenting cell by activating crucial signal proteins against cancer [28], endowing the nano-aAPCs the capacity to be one among the next generation cancer nanomedicine [1].

## 3. Mechanism of Action of Nanoparticles

**Figure 1:**
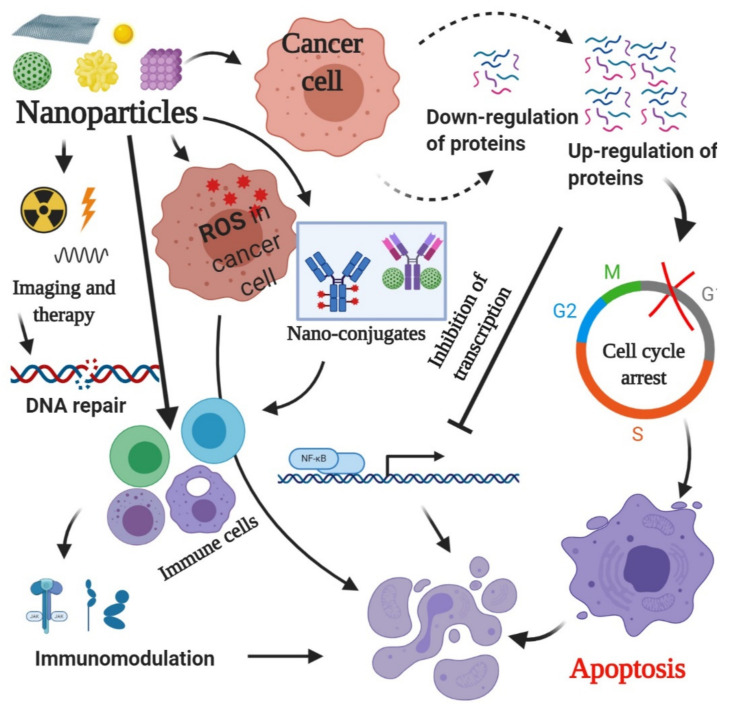
Mechanisms of cell death in cancer induced by nanoparticles.

Generally, nanoparticles induce apoptosis in cancer cells by a series of mechanisms; reactive oxygen species (ROS)-mediated apoptosis being the most studied among them. Up- and down-regulation of proteins, immunological interventions, inhibition of transcription, site-specific cytotoxicity, etc. are other mechanisms of action of nanoparticles in apoptosis induction in cancer cells. It is imperative to mention at this point that there is a series of cross-talk between many of these events but the final effect is apoptotic cell death (Figure 1).

### 3.1. Generation of ROS

ROS induced-apoptosis is one of the basic mechanisms of action that has been studied as part of nanoparticle-induced cytotoxicity. Generally, ROS behaves like a double-edged sword bringing about anti-apoptotic and pro-apoptotic effects. The pro-apoptotic effect of ROS sums up to cell cycle arrest, apoptosis, and necrosis whereas, the anti-apoptotic effect transpires to the promotion of cell proliferation, invasion, and metastasis all the while inhibiting apoptosis [29]. Proteins are capable of inducing ROS-mediated apoptosis. Fragile histidine triad (Fhit) proteins are lost in most cancer types but when restored, they can induce apoptosis. Fhit interacts with ferrodoxin reductase to trigger the generation of ROS in Fhit-deficient cancer cells following peroxide treatment. Oxidative stress is induced, and this resulted in ROS-induced apoptosis in lung cancer cells. It was observed that Fhit negative cells escaped ROS overproduction and, likely oxidative damage [30]. Nanoparticles are more effective in inducing apoptosis mainly since nano-sized particles offer more reactivity due to increased surface area resulting in the excessive formation of ROS. The apoptotic effects are prominent when oxidative stress increases for the cells and releases inflammatory intermediates followed by DNA and protein damage resulting in cytotoxicity [31]. Silver nanoparticles enclosed in polysaccharides were found to generate significant amounts of ROS which, in turn, educed cell death mainly through autophagy and extend apoptosis [32]. Nanoparticles targeted to mitochondria through pyruvate produce ROS resulting in the inhibition of ATP synthesis. Silica carbon nanoparticles coated with lipid membrane were found to inhibit the growth of multidrug-resistant tumors with no evidence of systemic toxicity through inhibition of ATP synthesis [33].

### 3.2. Regulation of Proteins

Up- and down-regulation of proteins represent a situation where the cells mimic a cellular response to any endogenous or exogenous stimuli under normal or oxidative stress conditions. Under stress, more proteins are regulated to remodel the metabolic and signaling pathways, leading to alterations in the redox proteome and membrane turnover, impacting cycle progression and proliferation, resulting in apoptosis and tumor suppression [34]. There is increasing evidence for the potential of nanoparticle-mediated regulation of proteins that are involved in signaling pathways specifically associated with the pathogenesis, progression, and oncogenic behavior of cancer cells. Copper oxide nanoparticles (CuO-NPs) are capable of the down-regulation of the apoptotic regulatory proteins (Bcl_2_ and Bcl_xL_) inducing programmed cell death in HT-29 under study [35].

Selenium nanoparticles (SeNP) were found to have different effects on the expression profile of apoptotic proteins at concentrations as low as 5 µg/mL. The unfolded protein response (UPR) signalling pathways were also affected by the SeNP. Treating cell lines with SeNP showed a significant increase (3–4 fold) in the expression pattern of ER-resident selenoproteins and selenium-containing glutathione peroxidases and thioredoxin reductases [36]. Pro-apoptotic proteins were also selectively regulated by SeNP by the activation of Cx43 hemichannels [37]. Likewise, enhanced expression of γ-H2AX was detected in MCF-7 cell lines that positively took up silver nanoparticles (AgNPs), followed by the release of Ag ions within the cells and subsequent cell death [38]. Selective regulation of cyclin dependent kinase 4 (CDK4) by gold nanoparticle conjugates induced the G1 cell cycle arrest and apoptosis induction in the ER-positive human breast cancer cells lines, MCF-7 [39]. The inactivation of CDK4 was followed by a failure in the translocation of nuclear factor kappa B (NF-κB) to the nucleus resulting in cell death. Gold nanoparticles could potentially down-regulate the phosphorylation of key players (like p42/44 and p38 in MAPK signaling pathway), thus inhibiting migration and colony-forming ability of pancreatic cancer cells and in turn, reverse the chemo-resistance in cancer cells [40].

### 3.3. Radiation Therapy

Traditional radiation therapy fails to achieve the desired effect in cancer therapy as indiscriminative radiation renders damage to normal, healthy cells too. Also, opsonization leads to the rapid clearance of radioisotopes from the blood resulting in reduced therapeutic effect. Employing nanoparticles with a radioactive core will reduce opsonization and also help in extending the retention of the therapeutic nanoparticle through enhanced permeability and retention (EPR) effect [41]. The use of nanoparticles (as radiosensitizer) for systemic targeting of tumor cells in radiation therapy will enhance the effect of X-ray radiation therapy against cancer cell lines. The biological hazard to other cells due to radiation can be controlled by enclosing the radioactive molecule in a nano-crystalline matrix, bound by strong crystalline bonds. This relatively new approach engages nanoparticles as sensitizers whereby, the nanoparticle with a radioactive core is attached to the molecule and can be precisely directed to the cancer cells. Upconversion nanoparticles (UCNP) coupled to beta-emitting radionuclide yttrium-90 (90Y) and a fragment of the exotoxin A that was genetically fused to a protein specific to HER2 receptor, enabled background-free imaging in in vitro and in vivo cell models [42]. The localization of radiation impacted only those cells within millimeters leading to the reduction of adverse side effects also. Radioactive palladium gold nanoparticles were efficiently retained in prostate cancer tumors for several weeks to deliver a dose-dependent tumor growth inhibition. The interaction of energetic photons with gold atoms produced photoelectrons, Auger electrons, X-rays and delta rays. These electrons damage the tumor and blood vessel endothelial cells causing DNA strand breaks resulting in cancer cell death [43]. Bismuth lipophilic nanoparticles effectively enhanced the X-ray radiation therapy against breast cancer cells [44], thus effectively inducing a concentration-dependent cell growth inhibition in breast cancer cell lines by possibly altering the membrane permeability and damaging the genomic DNA [45]. Sometimes, bismuth (Bi) nanoparticles are favored to gold (Au) or platinum (Pt), as they are readily oxidized and dissolved at physiological conditions. Also, the high atomic number and biocompatibility favored the use of bismuth in radiation therapy. They are released from the body as soluble ions and so, bioaccumulation is minimized. XCA (X-ray contrast agent) containing bismuth (like Bi_2_S_3_) nanoparticle coated with PVP (polyvinyl pyrrolidone) increased the computed tomography (CT) brightness, even at lower concentrations, as compared to the iodine-based clinical contrast agent, iopromide. The blood circulation half-life of PVP-coated Bi_2_S_3_ nanoparticles was significantly larger than the iodinated counterparts [46].

### 3.4. Phototherapy

**Figure 2:**
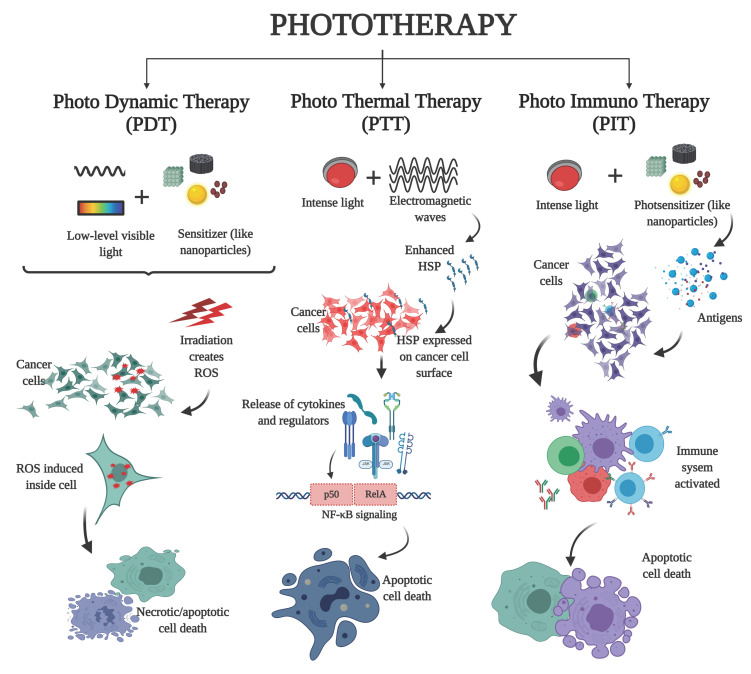
Phototherapy is used to induce apoptosis in cancer cells by employing light, heat, and radiation. Photosensitizers like nanoparticles enhance the killing effect by targeting signaling pathways and the immune system.

The heat generated from light or electromagnetic radiation is used to elicit responses in cancer cells that induced apoptotic cell death as in phototherapy (Figure 2). Also, in the presence of thermal stress, the radio-sensitized tumors are likely to respond even more to radiotherapy, resulting in improved cancer survival rates [47]. Nanoparticles act as the photosensitizer whereby, due to their higher optical absorbance in the near-infrared region, the light energy from the source is transmitted to the tumor site as thermal energy, resulting in the release of reactive oxygen species, immunogenic molecules, or antigens that are detected by the cell’s immune system and process the tumor cell for destruction by apoptosis [48]. Among various nanoparticles that are employed to deliver heat to the tumor cells, the iron oxide nanoparticle magnetite (Fe_3_O_4_), is capable of emitting thermal energy when exposed to a rapidly alternating magnetic field. The heat thus generated has a more discriminatory effect on fast-dividing cancer cells than normal tissues [49,50]. A single 10-min exposure of the oral cancer cell lines (VB6) to magnetic nanoparticles conjugated to anti-αvβ6 antibody in an alternating magnetic field resulted in the death of 85% of cell lines. Only a 20% reduction in cell viability was observed for the αvβ6-negative cells exposed to the same conditions [51]. Gold nanoparticles (AuNPs) have very strong absorption around infra-red regions of the electromagnetic spectrum and can generate localized heat to destroy the region of interest and can thus be successfully employed for in vivo phototherapy [52]. An Au conjugate (Au@ZIF-8 based on nano metal-organic frameworks) acted as catalase mimicking nanozyme when Ce6 was encapsulated as a photosensitizer. It was observed that upon arrival of Au@ZIF-8 at the tumor environment, the AuNP catalyzed the excessive H_2_O_2_ to mitigate hypoxia, resulting in toxicity in the tumor [53].

### 3.5. Triggering Immunological Reactions

Immunological reactions are often the most fundamental reactions that expose the tumor cells for eradication via the host immune system by tumor antigens [54]. Enhancing the immunity with biocompatible nanoparticles is a strategy successfully adopted to control cancer cell growth. Actually, cross-talk between the various processes (like ROS release and the subsequent complement activation) is evident as many key mechanisms in immunity are inter-connected. The release of cytokines when THP-1, NCI-H460, and HL-60 cell lines were treated with gold and silver nanoparticles, clearly implicated the involvement of humoral and cellular immunity upon exposure of the cells to the nanoparticles. This, in addition, provides a fine case for use of nanoparticles as an adjuvant in vaccine development as evidenced by the balanced Th1 and Th2 immune response mediated by ROS production, cytokine release, and complement activation [27]. Another strategy is to modulate the immunosuppressive microenvironment of the tumor and thereby kill the cancer cells by employing nanoparticle-encapsulated dual therapeutic molecules (like nucleic acid). Here, the dual molecule can be effectively engaged for the delivery and repeated administration of the high-dose nanoparticle-encapsulated nucleic acid, resulting in systemic immune activation followed by elevated levels of pro-inflammatory molecules [26]. The nanoparticle-encapsulated dual therapeutic 5′ triphosphate ds RNA (ppp dsRNA) induced higher levels of Th1 cytokines, CD8^+^ T cells, M1 macrophages, etc. reflecting significant tumor growth inhibition. Furthermore, the physicochemical and colloidal nature of nanoparticles enables the production or inhibition of antibodies, which endorse their extensive use in the diagnosis and treatment of melanoma [55].

### 3.6. Site-Specific Cytotoxicity

The customized drug release profile is one of the rationales behind the use of nanoparticles loaded with the drug conjugate to be targeted for site-specific release. Intracellular delivery of DNA, mRNA, small interfering RNA (siRNA), and protein can be facilitated by nanoparticles where the delivery of nano-conjugated biomolecules offers site-specific cytotoxicity with reduced side effects that are generally caused by systemic drug formulations. This enhanced therapeutic efficiency comes with fewer adverse effects, improved pharmacokinetics, higher EPR effect [56]. Moreover, the excellent binding efficiency of the target ligands on the nanoparticle surface leads to increased tumor selection and drug disposition in cancer cells. The negatively charged hyaluronic acid-based nanoparticles were found to have site-specific cytotoxicity towards CD44-positive tumor cells [57]. Also, the therapeutic efficacy can be monitored in real-time as is revealed by the targeted delivery of chemotherapeutic drugs by the multifunctional titanium phosphate nanoparticle. These were designed for cellular uptake and cytotoxicity as the nanoparticles exhibited high drug loading capacity and enhanced cell uptake mediated by folate-receptor [58]. In another typical case, zinc oxide (ZnO) as such was found to have inherent cytotoxicity towards cancer cells by inducing apoptosis. The high expression of membrane anionic phospholipids on the surface of cancer cells renders them the capacity to selectively intake ZnO nanoparticles. This leads to an imbalance in Zn ions resulting in the induction of ROS. Conjugation of ZnO with other metal oxide nanoparticles (like Fe_3_O_4_) is found to increase the cytotoxic potential because of the combined effect of ZnO selectivity and Fe_3_O_4_ magnetism [59]. The cationic solid lipid nanoparticle (SLN) nano-conjugate was able to tightly bind streptavidin and biotinylated antibody against HER2 receptor in breast cancer cell lines. These anti-HER2 compact antibodies acted as highly effective carriers and effected specific cell cytotoxicity in BT-474 and MCF-7 cell lines that had overexpressed HER2 [60]. 

### 3.7. Gene Therapy for Cancer Cell Growth Inhibition

Regulation of genes that are actively involved in various cellular processes in tumor cells by nanoparticles is another strategy to kill cancer cells. Genes like *STAT3, FGFRL1, HNRNPL, BCL2L1, ATF3, RAB5C, ANG, EIF3I, NKIRAS2, PIAS4, RRAS, NUCKS1, AKT1, SRC, PCBP2, EIF2C2, HRAS, CDC34, NFKB2, EIF4G1*, and *EIF5A* are controlled by Fe_3_O_4_ nano-powder in A549 cell lines thus inducing antitumor effect [61]. Many of these genes are overly expressed in cancer cells and are rightly identified as molecular markers. The fact that Fe_3_O_4_ nanoparticles can induce an anticancer effect on cancer-specific molecular markers can have greater implications in gene therapy.

Many more nanoparticles were successfully employed to demonstrate these major mechanisms to control cancer cell growth. Table 1 lists some of the nanoparticles employed in cancer cell studies and the mechanism of action.

## 4. Nanoparticles in Clinical Translation

**Figure 3:**
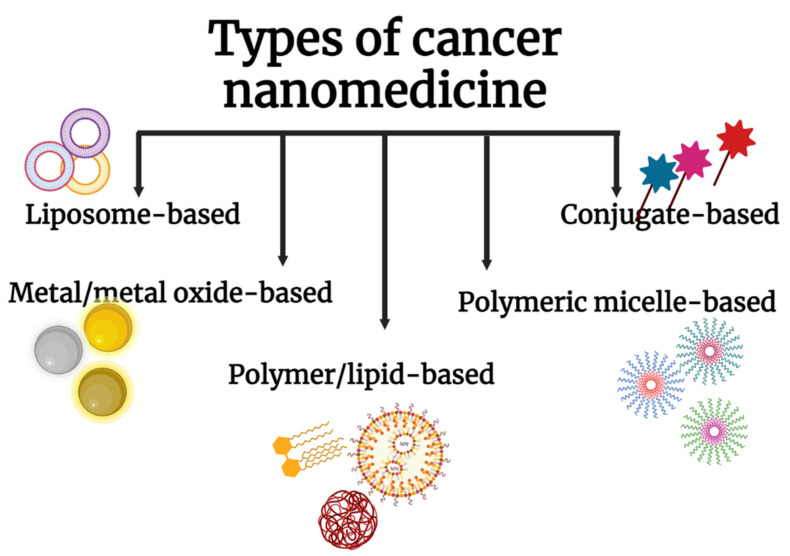
Different types of nanomedicines that are used for the treatment of cancer.

A google scholar search with the keywords ‘nanoformulations, cancer cure’ returned close to 3000 hits within 0.08 s; 18,000+ publications are based on nanomedicines. But it is upsetting from a researcher’s point of view to note that much of this basic research failed to get translated into clinical realities. 25 years after the first nanochemodrug Doxil was introduced to the market in 1995, the number of nanomedicines approved for cancer cure is still trifling. Despite the huge number of research attempts to use nanoparticles for cancer therapeutic applications, only very few formulations have crossed the clinical phase over the period. A fleeting look into the NP-based formulations currently on the market and in clinical trials will reveal that there are not many nano-formulations that have got the approval to hit the market as cancer therapeutics [83]. 16 nano-based cancer drugs are approved by FDA whereas close to 75 nanoformulations are in clinical trials now [84]. There are five major cancer nanomedicines available for cancer cure (Figure 3).

### 4.1. Liposomal Nanoparticles

**Figure 4:**
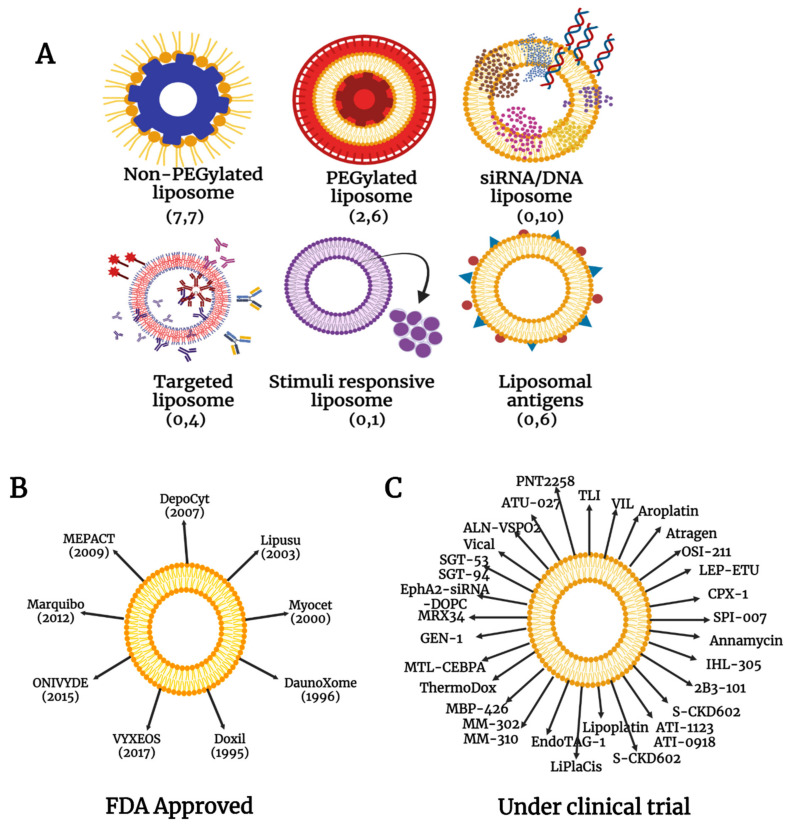
(**A**) Various forms of liposome-based nanomedicines. The variety of formulations under the category specifies the flexibility of the liposomes for the formulation of nanodrugs. The number in parenthesis refers to the number of drugs that are approved and under trial respectively, under each category. (**B**) Major FDA approved nanochemotherapeutic drugs based on liposomes. (**C**) Some of the liposome-based nanochemotherapeutic drugs that are under clinical trial.

Most of the nanoparticle-based cancer drugs are formulated with liposomes (Figure 4) as the liposome-based nanodrugs have greater adaptability and feasibility in addition to biocompatibility and biodegradability [85]. The exceptional ability of liposomes to entrap both hydrophilic and hydrophobic compounds along with their ability to be functionalized with a variety of molecules like PEG, antibodies, aptamers, proteins and peptides, carbohydrates, or other small molecules in targeted liposomes [86] may be the prime reason why the majority of approved drugs and those in clinical trials belong to this group. During drug delivery, the bilayered phospholipid membrane of liposomes protects the drugs embedded in them from proteasomal degradation and biological inactivation; they are resistant to chemical and immunological changes as well [87]. The major advantage is that the non-targeted, healthy cells in the vicinity of the drug target are unaffected by the drug carried by the liposomes as they are safely entrapped in the liposomal core.

### 4.2. Metal and Metal Oxide Nanoparticles, Polymeric Micelles, Polymer/Lipids, and Other Conjugates

**Figure 5:**
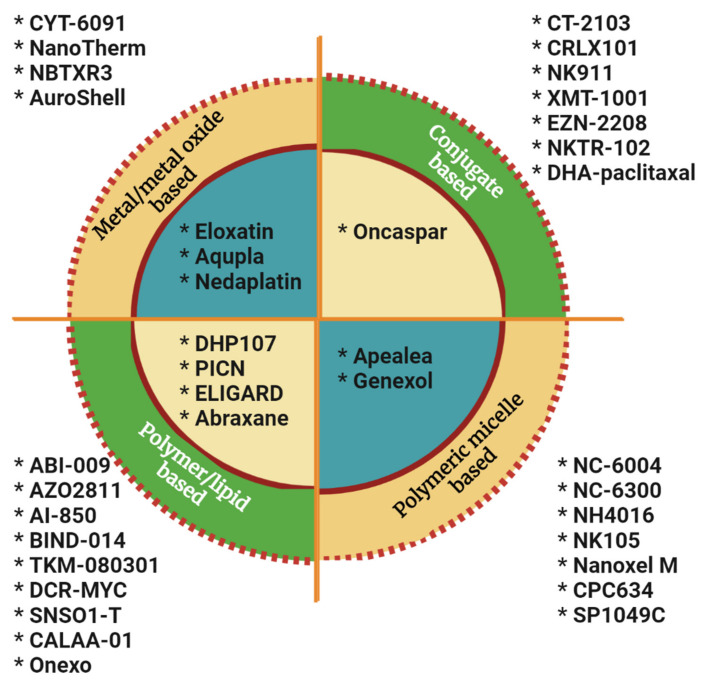
Nanochemotherapeutic drugs that are based on metal and metal oxides, polymeric micelles, polymer/lipids, and other conjugates. (The formulations inside the circle are approved and those outside are under trial).

The rest of the drugs are based on metal and metal oxides, polymeric micelles, polymer/lipids, and other conjugates (Figure 5) where the drugs are in various stages of approval or trials. Platinum is one of the most exploited metals for the delivery of anticancer drugs; cisplatin is the first to be used as an anticancer drug followed by a variety of other compounds. Platinol was approved by FDA for combination therapy for a variety of cancers in addition to picoplatin, carboplatin, sebriplatin, ormaplatin, oxaliplatin, aroplatin, enloplatin, satraplatin, zeniplatin, miboplatin, satraplatin, and iproplatin [88]. However, the dominance of platinum-based drugs was diminished during later years as a spectrum of challenges emerged from the use of platinum and platinum-based drugs in clinical use. This prompted the emergence of other nanocomplexes with cytotoxicity in cancer therapy (like ruthenium [89], gold [90], silver [91], selenium [92], and iron [93]). More and more metal complexes are being studied for antitumor effects based on the success of metal-based nanodrugs.

Vesicles formed from self-assembled amphiphilic micelles in the nano range can extravasate through endothelial cell junctions of cancer cells and thus can erupt near the tumor microenvironment to release the drug. These polymeric micelles are better in drug delivery as the internalization of the drug carrier to the tumor site is more effective than liposomes and other lipid nanoparticles [94]. Drug solubility is improved while core-shell structured polymeric micelles are used as drug carriers; the water-insoluble drugs are entrapped in the micelle core by physical or chemical conjugation promoting the availability of the drug to the site of action [95].

Exploiting the improvements in polymeric systems based on lipids, a new nanodrug system was developed in recent years. The lipid-polymer nanoparticles comprise a hybrid structure comprised of a polymeric core surrounded by a lipid shell [96]. They are postulated to incorporate the benefits of lipid-based nanocarriers including the easiness in drug release along with additional features of surface modification and functionalization.

In a nanoconjugate drug system, the various components are premeditated and designed using nanosheets as a delivery platform attached to a receptor targeting polymer (like folate receptor) [97]. New cytotoxic agents are designed to maximize the anticancer effect by targeting the tumor. The fatty acid Docosahexaenoic acid conjugated with the antineoplastic drug paclitaxel, (DHA–paclitaxel) is one such novel semisynthetic taxane conjugate with no cytotoxic activity until it is metabolized to paclitaxel [98]. The tested doses of the taxane-conjugated drug were shown to have a greater incidence of myelosuppression than conventional taxane in patients with advanced non-small cell lung carcinoma (NSCLC) implying significant clinical application.

## 5. Obstacles in the Clinical Translation of Nanoparticles

In spite of all these developments, there are major obstacles in the journey of nanodrug from clinic to market. The lack of proper understanding of the mechanism of action of the nanoparticle with the biomolecules is the foremost deterrent factor. In many cases, continuous production of nanoparticles with sustained effect on cancer cells becomes a liability to the pharmaceutical companies. There are many examples where pharmaceutical companies have discontinued drugs (e.g., DepoCyt). These companies cite technical issues as reasons for the impediment. Researchers and pharmaceutical companies have to confront a multitude of issues before the drug is promoted as suitable for cancer cure. Due to this, many of the nanoformulations were retracted from the market even after getting FDA approval; Clariscan (PEG-Fero, Feruglose NC100150); Feridex I.V. (Endorem, Ferumoxides); Lumirem (Gastromark); Resovist (Cliavist); Sinerem (Combidex) and GastroMARK are examples [99,100].

There are some reasons why these research findings are not decoded and marketed. Issues that can cause probable hitches in the process are varied. Some of the issues are discussed here.

### 5.1. The Difficulty in Predicting the Predisposal of the Patient to Allergic Reactions

It is very difficult to predict a definite pattern or behavior even in patients who are administered the same dose of medicine. Though the relevant mechanisms of action of the drug during the clinical study in a varied population of patients showed highly similar outcomes, they may have unexpected and contradictory side effects when it reaches the mass population of patients worldwide. Likewise, a simple assessment of side effects does not guarantee the safety of the drug [101] This in itself is a complex scenario as there is an infinite potential for the drug to combine with different entities that it confronts in the body and a methodical examination and assessment of this interaction at a molecular level are important.

### 5.2. Endotoxin Quantification

A common reason for the early failure of clinical translation of nanochemoformulations is endotoxin contamination [102]. The common assay for endotoxin quantification is the limulus amebocyte lysate (LAL) assay where the endotoxin’s activity is measured [103]. However, this assay fails to quantify endotoxin as the activity and not the endotoxin, is measured. Alternate methods like Endotoxin Activity (EA) assay and anti-endotoxin monoclonal antibodies were attempted but none of these were adequate to be administered beyond the trial period.

### 5.3. The Cellular Internalization of the Drug

A drug usually circulates and accumulates at the site of action before it is internalized in the cell. This process remains a chokepoint in the development of nanochemotherapeutic drugs mainly because of the inability of the drug to go inside the tumor cells [104]. Unlike conventional drugs, the full intracellular, paracellular, and transcellular pathways of the nanodrug across any biological membrane are not completely understood and therefore, cellular internalization of nanochemotherapeutic drug possess an obstruction to its clinical development. The pharmaceutical formulation will follow only after a complete characterization of the internalization route [105]. Strategic research to overcome this dilemma was carried out when a nanomedicine based on a polyprodrug was developed that has the ability for zwitterionic-to-cationic charge conversion [106]. These pH-responsive nanomolecules respond well to the tumor microenvironment. The surface charge conversion effected by the nanodrug improves the efficiency of cellular internalization of the nanomedicines. Redox-responsive polymeric micelle conjugates are developed lately to target proteins and thus help in releasing the loaded drug to the designated site against cellular internalization complexities [107].

### 5.4. Sustained Release

Exceptional and sustained delivery of drugs to the target organs with minimum side effects is often a problem when drug development is involved. The EPR effect is a deciding factor in drug release and delivery. In a recent approach, stimuli-responsive nanocarriers were developed where biological (internal) or physical (exogenous) stimuli were directed to specifically targeted cells after drug delivery and the precise release of the drug was achieved [108].

### 5.5. Overcoming Biological Barriers along with Increased Bioavailability

The accumulation of nanotherapeutic drugs at sites of disease progression is limited by biological barriers. Sometimes, the drug molecules merely get dispersed and distributed freely all over the body, resulting in detrimental side effects. This will also reduce the impact or response that the prescribed doses were supposed to bring about as expected by the physician [109]. The role of a suitable drug delivery system cannot be ignored at this point. Nanocarriers have brought about a revolution in the targeted drug delivery domain where delivery of nanochemodrugs that can traverse across different biological barriers was made possible. A recent article cited the use of *Prunus spinosa* fruits (PSF) containing different phenolic compounds as biomimetic nanoparticles that could improve the activity of the extract where the payload is directed into the lipid bilayer with increased accumulation at the diseased tissue (showing enhanced specific targeting properties). Improved biocompatibility, as well as a low toxicity, was observed when the nanoparticle was evaluated on HUVEC cells suggesting a unique platform for encapsulation of drugs that have minimal stability and bioavailability [110].

### 5.6. Increasing the Functional Capability to Target Only Tumor Cells

The heterogeneity exhibited by tumor cells and the relational differences among various tumor tissues inevitably contributes to the failure of drugs to target the tumor cells alone. It is a challenge to obtain any drug that is specific to different tumors and patients. The inconsistent characters of individual patients and their response to the drug contribute highly to the failure to target tumor cells alone. It is often difficult to select, purify and identify optimal ligands to carry the nanodrug. Nevertheless, natural cellular materials (like exosomes) with intrinsic targeting properties can serve as nanocarriers for a drug aimed exclusively at tumor cells [111].

### 5.7. Controlling Immune System Response to the New Drug

It is difficult to predict the response of the immune system to the advent of a new molecule in the system. This is a major reason why the current drug therapies can extend survival rates but not cure the disease per se. The human immune system is sophisticated and complex It would be a ‘dream-come-true’ situation if the mechanism could be deciphered properly. Products could be developed that could control the immune system to treat a wide variety of diseases. Immunotherapy is a novel cancer treatment centered around the manipulation of the immune system [112]. Here, a T-cell infusion system was developed that will reflect the mechanism mediated by the transplanted donor immune system rather than the host immune system. The mechanism involved generated the Mirror Effect™ that elicits a host vs. tumor (HVT) effect along with a non-toxic host vs. graft (HVG) response that was found effective in cancer therapy, among other diseases. Similar to this, innovative ideas could change future research in drug discovery if they are focused on utilizing and exploiting the mechanism whereby the human immune system eliminates cancer and other diseases. This could lead to potential curative outcomes that revolutionize the chemotherapeutic and pharmaceutic industries.

**Figure 6:**
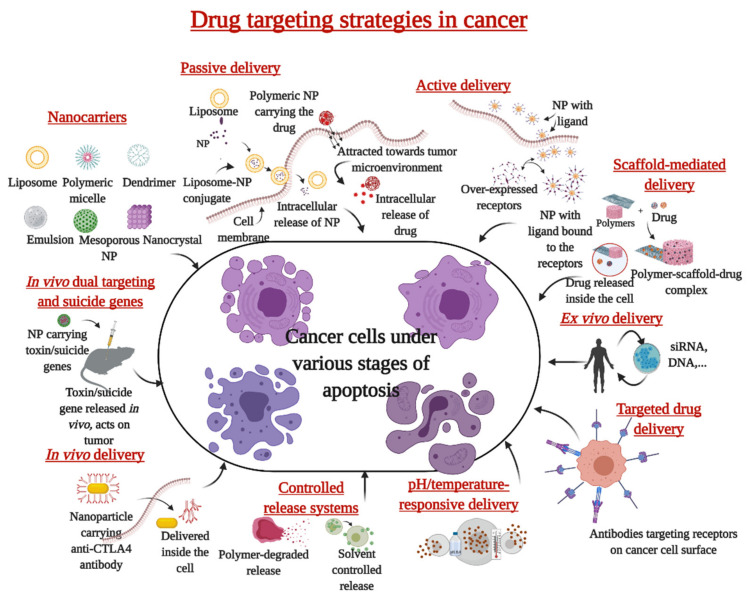
Schematic representation of the drug targeting strategies followed to deliver therapeutic molecules to cancer cells. The various modes of drug delivery ensure that the drug is effectively delivered to the cells of interest and thus, unwanted damage to normal cells can be minimized.

Other challenges in this process are enumerated in Table 2. An exemplary drug delivery system must selectively deliver the drug to a specific location in the body to increase its therapeutic efficacy. In the case of cancer therapeutics, the efficiency is measured in terms of the ability of the drug to induce apoptosis in cancer cells. How effectively the therapeutic molecule reaches the drug target with the minimal adverse effect is crucial here. Selective accumulation of drugs in tumors through EPR effect, poor bio-accessibility of the drugs to tumor tissues, inadequate cellular uptake leading to the requirement of higher doses are some of the concerns to be addressed for effective therapy. Higher dosage leads to elevated toxicity to normal cells and a possibility for multiple drug resistance. Recent research revealed that nanoparticles are exceptional in addressing these challenges while avoiding toxicity to normal cells [113]. They can be employed to effectively deflect the unwanted toxicity to normal cells all the while ensuring the efficient induction of cell death of cancer cells. Keeping the focus on inducing maximum damage to the cancer cells, different innovative targeting strategies are followed to deliver therapeutic molecules like nucleic acids, proteins, genes, small molecules, and monoclonal antibodies to cancer cells. Nanoparticles are being established as the best option for deploying drugs through various delivery routes. Some of these delivery routes are schematically represented in Figure 6.

## 6. Cutting-Edge Developments in Nanochemotherapy

Developments in cancer therapy are never-ending and with advanced tools and techniques, more and more robust systems are evolving. These could be solutions to overcome the obstacles encountered in cancer therapy. Some of the recent developments are briefed as below.

### 6.1. CRISPR—The Gene-Editing Tool

Clustered Regularly Interspaced Short Palindromic Repeats (CRISPR), the Nobel prize-winning discovery developed by Jennifer Doudna and Emmanuelle Charpentier, is a gene-editing tool with high precision. This could alter human DNA so as to eliminate chances of disease incidence. CRISPR is the immune model that is used by bacteria to prevent invasion by bacteriophages. At its core, the CRISPR system enables prokaryotes to accurately recognize genetic sequences of phage and thus earmark these sequences for further destruction using unique enzymes. CRISPR/*Cas9*, a variation of technological innovation, is used for the treatment of cancer, where multiple genetic alterations are established [126]. Many recent researches successfully employed nanoparticles and nanoformulations to promote CRISPR/*Cas9* development [127]. The antibody-conjugated tumor-targeted nanolipogels (tNLGs), were employed to efficiently encapsulate the CRISPR plasmids independent of electrostatic interaction, thus eliminating the cationic toxicity [128]. The highly efficient tNLGs utilize an antibody-guided strategy to selectively recognize and bind cancer cells, especially triple-negative breast cancer (TNBC) while sparing normal tissues, the low particle elasticity of the nNLGs allows them to directly release CRISPR plasmids into the cytosol of targeted TNBC cells via a receptor-mediated membrane fusion pathway, effectively avoiding endosome entrapment within TNBC cells. Lipid nanoparticles formulated with biodegradable ionizable lipids, PEG-DMG, Spy Cas9 mRNA, and sgRNA can effectively deliver CRISPR/*Cas9* components in vivo for genomic editing and produce sustainable gene knockout within 52 weeks after a single administration [129]. Further, researchers have developed a multistage delivery polymer nanocarrier (MDNP) that responds to the tumor’s slightly acidic environment, thus achieving tumor-targeted delivery of the CRISPR/*Cas9* system. MNDP can overcome various physiological barriers and achieve targeted delivery of CRISPR/*Cas9* to inhibit tumor growth [130]. Cell type-specific targeting using nanoparticles that encode DNA aptamers are used to target specific tumor cells. A stimulus-responsive *Cas9/sgRNA* release with enhanced genome editing efficiency was achieved when they were combined with DNA nanoflowers [131]. The Cas9 protein (chemically modified with a glutamate peptide tag at the N-terminus) and sgRNA were delivered using cationic arginine-functionalized gold nanoparticles with an efficiency as high as 90–95% [132].

### 6.2. ThermoResponsive-NanoVelcro Purification System

This is a nanomaterial-embedded cancer diagnostic platform expected to bring together a distinct ‘rare-cell’ sorting method that enables detection, isolation, and characterization of circulating tumor cells in the peripheral blood. This is envisaged to provide non-invasive monitoring of the disease progression in patients. Previously, a nano Velcro chip was designed with PLGA embedded into it to capture M229 cells with high precision (87%) [133]. Another study was done where the NanoVelcro CTC purification system was used along with the NanoString Counter platform for cellular purification and RNA analysis in metastatic castration-resistant prostate cancer [134]. This mode of tumor therapy facilitates cancer patient care and has the advantage of early detection of drug resistance.

### 6.3. PROTAC—A Novel Proteolysis Targeting Entity

As every disease is caused due to the disparity of the associated protein moiety, finding novel drugs that can accurately control protein synthesis and degradation is an alternate approach. Dysfunctional proteins are the major reason for cancer and a drug that can inhibit the activation of such abnormal proteins and their subsequent pathways will be valuable for cancer cure. Proteolysis Targeting Chimeras (PROTACs) are emerging groups of small molecules that can effectively remove the dysfunctional proteins from the body [135]. These heterofunctional molecules can hijack the body’s natural disposal system and specifically choose the concerned protein to degrade them. Only very small amounts of PROTACs are needed for the action as compared to other conventional drugs, this reduces the possibility of the adverse effect.

BRD4 is a protein belonging to the Bromodomain and Extra terminal (BET) protein family. It acts as super-enhancers (SEs) organizer and a regulator of oncogenes’ expression [136]. The development of ARV825, a BRD4-degrading PROTAC molecule with anticancer activity was reported [137]. Authors report that the nanoformulation of ARV acts as a selective degrader of the BRD4 protein and is effective in targeting the ‘undruggable’ *c-Myc* for the treatment of pancreatic cancer. In another study, gold nanoparticle-based multiheaded PROTAC molecules were found effective in the targeted degradation of anaplastic lymphoma kinase, a major target in treating NSCLC [138]. Vectorization of encapsulated BET-PROTACs with antibody-conjugated nanoparticles (ACNPs) facilitated their controlled release and enhanced their pharmacokinetic and efficacy profile. In this study, the commercially available MZ1 PROTAC (a selective degrader of BRD4) was encapsulated into the FDA-approved polymeric nanoparticle—trastuzumab—to guide the delivery of MZ1 to breast cancer cells that overexpress HER2 [139].

### 6.4. Proton Therapy—An Alternate Approach to Conventional Radiation Therapy

Scientists at the Paul Scherrer Institute in Switzerland launched a novel alternative to conventional radiotherapy when they introduced proton beam therapy with the hope that it will prolong the survival rates of lung cancer patients. The photon beam therapy is expected to combat and reduce the adverse impact of radiotherapy or the conventional megavoltage (MV) photon therapy [140,141]. Proton beam, which is charged particle that is directed to the tumor with high precision. The Proton can pass the entire tumor and deposit energy on the tumor. Radiation therapy is known to induce tumor death by producing DNA lesions in the tumor cells, mainly as single-stranded breaks (SSB) and double-stranded breaks (DSB) [142]. Conventional radiation therapy is given with curative intent in fractions of 1.8–2.0 Gy daily whereas only 1 Gy is needed for proton therapy to produce 1 × 10^5^ ionization events per cell. Around 1000–2000 SSBs and 40 DSBs are produced in this case, increasing lethality to tumor cells. Researchers predict a potential improvement in treatment outcome in cancer therapy when the dosimetric advantage of proton beams is integrated with radiosensitizers like gold nanoparticles [143] of intermediate size [144]. A functionalized hafnium nanoparticle (NBTXR3) was developed and clinically tested where it acted as a selective radioenhancer to kill the tumor cells exposed to radiotherapy by the absorption and deposition of high radiation dose within the cells [145]. The nanoparticles were activated by intensity-modulated radiation therapy (IMRT) and used in the treatment of advanced-stage head and neck squamous cell carcinoma (NHSCC).

### 6.5. Functionalized DNA—A Programmable Way to Deliver Cancer Therapeutics

DNA nanotechnology is a technique of recent origin where the strands of self-assembling DNA hybridize with each other and can be fabricated into a functional nanostructure with extremely high spatial programmability. Inorganic nanomaterials (2D nanosheets, fluorescent nanomaterials) were incorporated into the DNA strands to be applied for diagnosis and cancer therapy [146]. Further, different linear DNA strands with predesigned sequences were successfully hybridized to self-assembled DNA tiles to form uniquely shaped nanostructures [147]. These DNA tiles can be programmed to combine with other tiles to make 2D and 3D nanoribbon lattices. They can further be functionalized with other nanoparticles like gold [148], graphene or graphene oxide [149] so as to interact with receptors on the cancer cell surfaces to activate signal transduction or other cellular mechanisms.

### 6.6. Avatar—A Real-Time Data Based Translational Therapeutic Approach

Advances in translational medicine have reached a new phase where real-time investigations are varied based on user rendition of the disease and their experience with the current therapy. Avatar is an attempt where digital customization of the patient’s perception is demonstrated by innovative algorithms. These algorithms are specifically created to decide and predict clinical outcomes and the pros and cons of a specific field of research. These could be perceived as a digital twin of the patients’ identity wherein each and every treatment-related aspect like prognosis, treatment outcomes, complications, diagnostic operations, etc. could be virtually tried before execution upon the patients’ prompting [150].

### 6.7. Protein Catenation—A Novel Approach to Develop Artificial Antibodies

Catenanes are an interesting group of molecules with unique properties like enhanced thermal stability, remarkable proteolytic resistance and even enhanced enzymatic activity [151]. Catenation is an attractive approach to alter protein topology without much change in their native form. Exploiting the catenation process, it is now possible to develop new artificial antibodies that have a greater affinity to the target, better in vivo stability (due to resistance to proteolysis) and prolonged circulation time. These offer a more positive effect on the target molecule [152]. The higher-order protein catenation framework supports the insertion of various proteins of interest (POIs) like affibody (high-affinity proteins) specific to human epidermal growth factor receptor-2. This provides a good candidate for designing multivalent protein structures and motifs that can promote structure-activity relationships to advance protein therapeutics.

### 6.8. Other Approaches

A few other approaches were also evolved in recent times. The development of drug mimetics where a drug-like molecule can thwart the cancer-inducing effects of mutated regulator proteins [153,154], targeted radionuclide therapy (TRNT) where the radionuclides can potentially destroy tumor cells even if they do not possess specific tumor-associated antigen or receptor or biomarkers [155,156], bacterial cancer therapy where magnetically responsive bacteria are directed to tumor sites where they secrete toxins and compete for nutrients with the tumor cells thus destroying the tumor cells by modulating immune responses [157], multi-ion radiotherapy where a pure beam of heavy ions (like carbon ion) is used for radiotherapy [158], etc. are some of the other recent developments being explored for potential therapeutic uses in cancer cure.

## 7. Recent Advances in Clinical Studies with Nanoparticles in Tumor Therapeutics

As discussed earlier, bringing out a drug for chemotherapy to market is a race against time. In this era of precision medicine, a better understanding of translational research on where we stand and where we are going will help in designing a proper treatment regime for the various types of cancer.

Immunotherapy is one area where many advances have happened in terms of nanochemodrugs. The discovery of cellular and humoral immune responses to over-expressed tumor-associated antigens (TAA) like MUC1 in many patients with breast cancer and other forms of adenocarcinoma opened up many targets for immunotherapy. Tecemotide, a MUC1-specific cancer immunotherapy, is evaluated in Phase III clinical trials for the treatment of stage IIIA/IIIB NSCLC [159]. Similarly, Lipovaxin-MM, a dendritic targeted liposomal vaccine entered the phase 1 trial for malignant melanoma [160]. CRLX101 is a first-in-class nanopharmaceuticals based on cyclodextrin polymeric nanoparticle (CDP) technology that has the potential to translate therapy into clinical outcomes [161]. A combined polymeric nanoparticle consisting of a recombinant protein and cholesteryl hydrophobized pullbulan (CHP) complex was administered as repeated doses of the vaccine IMF-001, to patients with solid tumors that express NY-ESO-1 antigen. In other trials, combining the vaccine with PD-1 blockade held promise in human trials [162]. Further, many nanochemodrugs were evaluated for clinical trials in conjugation with protein components like albumin. The drug-binding capacity of the nanoparticle-conjugated albumin (Nab) complex is high and that is one of the reasons why most nanochemodrugs belong to this category. The therapeutic capacity of Nab-paclitaxel along with gemcitabine [163], atezolizumab [164], and cyclophosphamide [165] are being tried against metastatic and early-stage breast cancer. ABI-007 is another Nab-paclitaxel combination where a clinical trial was completed for stage IV NSCLC and metastatic breast cancer. Table 3 represents some of the nanoparticles in various forms that have undergone clinical study for different types of cancer.

## 8. Therapeutic vs. Diagnostic Nanoparticles

The current review was mainly focusing on the therapeutic applications of nanoparticles. Diagnostic nanoparticles also contribute substantially to cancer patient management. Diagnosis in cancer heavily depends on spectroscopic techniques; Raman spectroscopy (Raman scattering) being the most used one. The penetration power and acquisition speed can be boosted with high specificity and sensitivity on integrating nanoparticles as contrast agents in imaging [166]. Surface-Enhanced Raman Scattering (SERS) coupled with nanoparticles have many advantages like simultaneous detection of different types of cancer [167], and a possibility to visualize extended tumors in vivo. In an interesting development, scientists have come up with a smartphone-based device called Krometriks for the colorimetric detection of microRNAs (miRNAs). The inventors claim that Krometriks is an easily accessible and affordable miRNA diagnostic tool that can be applied in low-resource situations [168]. The assay (called plasmonic coupling interference—PCI), is employed for miRNA detection using SERS by integrating silver nanoparticles (AgNP). Another advanced method was developed recently where the ROS-responsive tripeptide sequence of Arg-Gly-Asp (RGD) was modified by nanoparticles for the simultaneous detection of cancer by near-infrared (NIR) imaging and photothermal therapy (PTT) [169]. Nanoparticles, especially the magnetic iron oxide nanoparticles, can act as coupling agents that bind to specific ligands that can detect gene mutations. This high-throughput screening method is highly sensitive and selectively detects different mutated nucleic acid sequences and can be employed for the diagnosis of cancer biomarkers [170].

## 9. Restrictions on the Use of Nanoparticles in Medicine

Though nanoparticles are generally synthesized and used for medicinal and therapeutic uses, the long-term side effects and drawbacks of using nanoparticles in medicine are still unknown [171]. Extensive studies are conducted on the toxicological aspects of engineered nanoparticles; concerns regarding the impact of these nanoparticles on public health are on the rise. Researchers believe that the extended exposure to nanoparticles can cause damage due to their superior penetrating power and unique toxicity due to ‘nano’size [172]. An extensive study on the developmental and neurobehavioral efficacy of nanoparticles is needed to address this problem. Unfortunately, due to the relative newness of the involvement of nanoparticles in the field of medicine, there is no efficient regulatory technique for the validation of the hazards associated with the use of specific engineered nanoparticles as a medicine.

## 10. Future Perspective

Nanoparticles are rapidly changing the direction of drug treatment. Incorporating the enhanced properties of nanoparticles to cancer diagnosis and therapy has opened new avenues. Passive or active nanostructures can be created to specifically target a drug to remote regions of the body that are inaccessible for normal macromolecular drugs. For example, the novel fiber-optic dosimeter (nanoFOD) device based on nanomaterial is used to pinpoint and measure the in vivo radiation dose in real-time that was given during external beam delivery in radiation therapy sessions [173]. Furthermore, nanorobotics and molecular nanosystems can create artificial organs and system mimics have the potential to control the future of nanochemotherapy. The ‘safe-by-design’ concept for nanomaterials that is currently being investigated by scientists is expected to offer pharmaceutical companies a cost-effective platform in risk management. The safety or risk assessment can be made early during the development stages and can be incorporated into a final product.

## 11. Conclusions

Many promising strategies were postulated and investigated in the past few decadesin fighting the cancer. Several unconventional methods lead to the incorporation of nanoparticle-based drugs in the treatment and care of patients. Still, there are challenges and blockages, including those related to regulations and approvals, that may slow down the progress. Nevertheless, it is hoped that the scientific community, with the joint participation from academics, regulatory agencies, and industrial partners, will be able to bring out new, patient-centric nanodrugs that will accelerate their journey from bench to bedside, thus providing a paradigm shift from conventional tumor therapeutics.

## Figures and Tables

**Table 1:** The variety of nanoparticles employed in cancer cell death and their mechanism of action.

Type of Nanoparticles/Nano-Conjugates	Cell Lines	Mechanism of Action	Reference
DNA-modified magnetic NPs	MCF-7	Suppression of RNA marker	[25]
Au, Ag NPs	Human peripheral blood mononuclear cells (hPBMCs)	Compliment activation, cytokine production	[27]
Gold NP-tagged toxin	MCF-7	Down-regulation of CDK-4 and MAPK	[39]
Au@ZIF-8 NPs	EMT-6 murine breast cancer cell	ROS generation	[53]
Fe_3_O_4_@AuNC@erlotinib	PANC-1	Selective targeting of overexpressed EGFR	[62]
GOx and PDA functionalized iron oxide NPs	MDA-MB-231, MCF-10A and 4T1	Photothermal therapy and ROS-mediated damage	[63]
V_2_O_5_	B16F10, A549, and PANC1	ROS-induced apoptosis	[64]
Fe_3_O_4_	HepG2	ATP-citrate lyase-dependent RAS signaling	[65]
Fe@Fe_3_O_4_@heparin	4T1 breast tumor cell line, HUVEC cell	ROS generation	[66]
PEGylated rhodium nanodots	CT-26 colon tumor	Down-regulation of TNF-α and IL-6	[67]
Au NPs	B16 melanoma cell	Up-regulation of Caspase 3, Caspase 9, Bid, Bax and down-regulation of BCl_2_	[68]
Au NPs-PEG-RNase A conjugate	SW-480	ROS generation	[69]
Au NPs	B16 F10 melanoma cell	Mitochondrial pathway-mediated apoptosis	[70]
RBC membrane-coated PLGA NPs	Pancreatic ductal adenocarcinoma	Tumor microenvironment modulation	[71]
PEGylated ZnO NPs	PANC1	ROS-induced apoptosis	[72]
ZnO NPs	Human acute monocytic leukemia cell line (THP-1)	Mitochondrial membrane damage and elevated ROS concentration	[73]
Ag NPs	HeLa	SubG1 arrest and apoptotic/necrotic cell death	[74]
Pt NPs	A549	Induction of apoptosis and cell cycle arrest	[75]
TiO_2_ NPs	LL2 mouse lung cancer cell line	Oxidative stress and cytokine induction	[76]
MoS_2_ nanoflakes	MDA-MB-231	Selective ROS generation and photo thermal therapy	[77]
Pt NPs	Human foreskin fibroblast cell	Damage to DNA and inhibition of DNA replication	[78]
CeO_2_ NPs	Mouse fibrosarcoma cell line	ROS-induced apoptosis	[79]
CeO_2_ NPs	A549	ROS-mediated apoptosis	[80]
ZnO NPs	MCF-7	Up-regulation of caspase-8 and p53	[81]
TiO_2_ NPs	HepG2, A549, MCF-7 and IMR-90	Oxidative stress	[82]

**Table 2:** Challenges in the clinical translation of nanoparticles in cancer therapeutics.

Challenges	Reference
The long process of drug development	[114]
Years required for pre-clinical and clinical research on higher animals and humans	[115]
Hassles in obtaining regulatory approval to release the drug in the market	[116]
Failure to effectively load the drug inside the nanoparticles	[117]
Instability of the formulation	[118]
Issues with biocompatibility and toxicity	[119]
Insufficient residence time in the body	[120]
Failure of the drug formulation to selectively accumulate on the target	[121]
Failure in loading, internalization, and drug release	[122]
Incomplete biodegradation and elimination	[123]
Challenges in cellular uptake	[124]
Failure to translate the in vitro results to in vivo studies	[125]

**Table 3:** Some of the nanodrugs that have undergone clinical studies in recent years (data compiled from clinicaltrials.gov).

Nanodrug	Conventional Drug	Cancer Type	Clinical Trials.gov Identifier
Paclitaxel Nab	5-Fluorouracil, Epirubicin, Cyclophosphamide (FEC)	Breast cancer	NCT00110695
	Carboplatin, Erlotinib hydrochloride	NSCLC	NCT01928160
	Phenelzine sulfate	Metastatic breast cancer	NCT03505528
	Doxorubicin hydrochloride, Cyclophosphamide, Filgrastim, Trastuzumab	Estrogen receptor-positive Breast cancer HER2-positive breast cancer	NCT00407888
	Bevacizumab, Gemcitabine hydrochloride	Breast cancer	NCT00623233
	Carboplatin, Erlotinib hydrochloride	NSCLC	NCT00661193
	Sargramostim	Brenner tumor, Fallopian tube cancer, Ovarian clear cell cystadenocarcinoma, Ovarian epithelial cancer	NCT00466960
	PIPAC	Peritoneal carcinomatosis, Ovarian cancer, Breast cancer, Stomach cancer, Pancreatic cancer	NCT03304210
	Carboplatin, Herceptin^®^	Breast cancer	NCT00093145
	Ceritinib, Cisplatin, Gemcitabine hydrochloride	Advanced malignant solid neoplasm, ALK positive lung cancer, Metastatic pancreatic adenocarcinoma, Stages III and IV of pancreatic cancer	NCT02227940
	Azacitidine (Vidaza)	Advanced or metastatic Breast cancer	NCT00748553
	Etrumadenant, IPI-549, Pegylated liposomal doxorubicin (PLD)	Triple-negative breast cancer, Ovarian cancer	NCT03719326
	Mifepristone	Male breast cancer, Recurrent breast cancer	NCT01493310
	Cetuximab, IMRT (Intensity-modulated radiation therapy)	Head and neck cancer	NCT00736619
	Cetuximab, Cisplatin	Head and neck cancer	NCT00833261
	Leucovorin calcium, Irinotecan hydrochloride, Fluorouracil	Adenocarcinoma, Cholangiocarcinoma, Gallbladder carcinoma, Gastric adenocarcinoma, Malignant gastrointestinal neoplasm, Metastatic pancreatic adenocarcinoma, Pancreatic adenocarcinoma, Stage III Ampulla of vater cancer, Stage III Pancreatic cancer, Stage IIIA Gallbladder cancer, Stage IIIA Gastric cancer, Stage IIIB Gallbladder cancer, Stage IIIB Gastric cancer, Stage IV Ampulla of vater cancer, Stage IV Gallbladder cancer, Stage IV Gastric cancer, Stage IV Pancreatic cancer	NCT02333188
	Imiquimod	Male breast cancer, Recurrent breast cancer, Skin metastases, Stage IV breast cancer	NCT00821964
	Lapatinib	Neoplasms, breast cancer	NCT00650910
	Pembrolizumab, Epirubicin, Cyclophosphamide	Malignant neoplasm of breast	NCT03289819
	Alisertib	Adenocarcinoma, Pancreatic neoplasms	NCT01677559
	Lapatinib	Bladder cancer, Brain and central nervous system tumors, Breast cancer, Esophageal cancer, Extragonadal germ cell tumor, Gastric cancer, Lung cancer, Ovarian cancer, Prostate cancer	NCT00313599
	Doxorubicin, Cyclophosphamide, Carboplatin, Trastuzumab, Bevacizumab	Breast cancer	NCT00254592
	BBI608, Gemcitabine, Oxaliplatin, Leucovorin, Irinotecan, Fluorouracil, MM-398	Metastatic pancreatic adenocarcinoma	NCT02231723
	Gemcitabine, Capecitabine	Pancreatic neoplasms, Pancreatic cancer, Adenocarcinoma	NCT01161186
	CORT125134	Solid tumors	NCT02762981
	Pembrolizumab	Metastatic urothelial carcinoma	NCT03464734
	Bevacizumab, Carboplatin, Temozolomide	Melanoma (skin)	NCT00626405
	Docetaxel, Ixabepilone, Paricalcitol	Breast cancer	NCT00637897
	Paclical^®^, Taxol^®^	Epithelial ovarian cancer, Primary peritoneal cancer, Fallopian tube cancer	NCT00989131
NC-6004 (NP-cisplatin)	Gemcitabine	Solid tumors	NCT02240238
CRLX101 (cyclodextrin-based polymer)	Camptothecin	NSCLC, Primary peritoneal cancer	NCT01380769
CPC634 (CriPec^®^)	Docetaxel	Ovarian cancer	NCT03742713
AGuIX	Polysiloxane gadolinium-chelates based nanoparticles	Brain metastases	NCT02820454
Docetaxel-PNP	Taxotere	Solid tumors	NCT02274610
VYXEOS	Cytarabine, daunorubicin	Acute myeloid leukemia	NCT04920500
ONPATTRO	Patisiran	Transthyretin amyloidosis	NCT03862807

Abbreviations: NSCLC—Non-small cell lung cancer, HER2—Human epidermal growth factor receptor—2, ALK—Anaplastic lymphoma kinase.
